# Development of a Simple Method to Detect the Carbapenemase-Producing Genes *bla*_NDM_, *bla*_OXA-48-like_, *bla*_IMP_, *bla*_KPC_, and *bla*_VIM_ Using a LAMP Method with Lateral Flow DNA Chromatography

**DOI:** 10.3390/diagnostics14101027

**Published:** 2024-05-16

**Authors:** Kei Mikita, Moe Tajima, Anwarul Haque, Yasuyuki Kato, Satoshi Iwata, Koichi Suzuki, Naoki Hasegawa, Hisakazu Yano, Tetsuya Matsumoto

**Affiliations:** 1 Department of Infectious Diseases, Keio University School of Medicine, Tokyo 160-8582, Japan; moetajima@keio.jp (M.T.); n-hasegawa@z8.keio.jp (N.H.); 2 Department of Infectious Diseases, Graduate School of Medicine, International University of Health and Welfare, Narita 286-8520, Japan; kmahaque@iuhw.ac.jp (A.H.); katoy@iuhw.ac.jp (Y.K.); tetsuya.m@iuhw.ac.jp (T.M.); 3 Department of Microbiology, Tokyo Medical University, Tokyo 160-8402, Japan; iwata.satoshi.8m@tokyo-med.ac.jp; 4 Department of Clinical Laboratory Science, Faculty of Medical Technology, Teikyo University, Tokyo 173-8606, Japan; koichis0923@med.teikyo-u.ac.jp; 5 Department of Microbiology and Infectious Diseases, Nara Medical University, Nara 634-8522, Japan; yanohisa@naramed-u.ac.jp

**Keywords:** loop-mediated isothermal amplification (LAMP), DNA chromatography, carbapenem resistance, carbapenemase-producing genes

## Abstract

Infections by carbapenemase-producing *Enterobacterales* constitute a global public health threat. The rapid and efficient diagnosis of *Enterobacterales* infection is critical for prompt treatment and infection control, especially in hospital settings. We developed a novel loop-mediated isothermal amplification (LAMP) method combined with DNA chromatography to identify five major groups of carbapenemase-producing genes (*bla*_NDM_, *bla*_OXA-48-like_, *bla*_IMP_, *bla*_KPC_, and *bla*_VIM_). This method uses DNA–DNA hybridization-based detection in which LAMP products can be easily visualized as colored lines. No specific technical expertise, expensive equipment, or special facilities are required for this method, allowing its broad application. Here, 73 bacteria collections including strains with carbapenemase-producing genes were tested. Compared to sequencing results, LAMP DNA chromatography for five carbapenemase-producing genes had a sensitivity and specificity of 100% and >97%, respectively. This newly developed method can be a valuable rapid diagnostic test to guide appropriate treatments and infection control measures, especially in resource-limited settings.

## 1. Introduction

Antimicrobial resistance (AMR) is an imminent threat to global public health. In 2017, carbapenem-resistant *Enterobacterales* (CRE), carbapenem-resistant *Pseudomonas aeruginosa*, and carbapenem-resistant *Acinetobacter baumannii* were ranked in the highest priority category of the World Health Organization (WHO) global priority list of pathogens [1]. If no urgent actions are taken, AMR will cause an estimated loss of 10 million lives and a USD 100 trillion loss in global production by 2050 [2].

Carbapenems are members of the *β*-lactam class of antibiotics that have long been considered to be last-resort drugs for treatment of multidrug-resistant Gram-negative pathogens [3]. CRE are defined as *Enterobacterales* that are resistant to any carbapenems including imipenem, meropenem, and ertapenem. Carbapenemases expressed by these bacteria fall into three classes that are defined according to their amino acid sequence: Ambler class A (serine carbapenemases), class B (metallo-β-lactamase), and class D (oxacillin (OXA)-type carbapenemases). The rapid emergence and spread of carbapenemase-producing strains is mainly caused by epidemics of bacteria bearing plasmid-mediated *Klebsiella pneumoniae* carbapenemase (KPC) (class A), New Delhi metallo-β-lactamase (NDM), imipenemase (IMP) and Verona integron-mediated metallo- β-lactamase (VIM) (class B), and OXA-48-like (class D) enzymes [4].

Carbapenemase-producing *Enterobacterales* (CPE) pose a global health threat due to their rapid dissemination that is enabled through the horizontal transfer of plasmids carrying carbapenemase genes [5]. Resistance to carbapenems mediated by carbapenemases is of high clinical, therapeutic, and epidemiological relevance. Due to the limited options to treat infections caused by these bacteria, CPE are often associated with hospital outbreaks as well as increased mortality and morbidity in infected individuals [6]. Therefore, the precise diagnosis of CPE infections and a comprehensive understanding of the epidemiological distribution of CPE among hospitals are fundamental for optimizing antimicrobial therapies and for developing effective infection control strategies. The global epidemiology of CPE exhibits large geographic variations, and thus, the continual monitoring of local prevalence is also essential for decisions concerning treatments and infection control [5,7].

Directly identifying and detecting carbapenemase-producing genes in *Enterobacterales* remains the gold standard for identifying CPE [3]. However, AMR affects all countries, and the burden is disproportionately higher in low-income and middle-income countries [2]. Currently carbapenemase genotypic testing is performed at relatively few institutes, but such testing should be expanded to evaluate most, if not all, clinical samples to optimize treatments and maximize control [8]. However, the equipment required for genotypic assays, including nucleic acid amplification technologies, gene hybridization and whole-genome sequencing, is expensive. Moreover, these techniques are complex and require specialized expertise that is often in short supply in resource-limited countries. Thus, developing a rapid, simple, and reliable detection method for identifying CPE is crucial for appropriate antimicrobial therapy, infection control, prevention, and surveillance, especially in low- or middle-income countries where the disease burden related to CPE is often high.

Loop-mediated isothermal amplification (LAMP) is a gene amplification method technique that requires no expensive equipment like a thermocycler since DNA amplification occurs at a constant temperature [9]. LAMP is often recommended for use in resource-limited areas due to its simplicity, rapidity, sensitivity, and use of low-cost equipment. However, a significant shortcoming of LAMP is a lack of easy-to-implement, sequence-dependent detection techniques that allow the multiplex detection of different target molecules [10]. Identifying standard techniques for real-time detection during LAMP, such as fluorescent intercalating dyes, turbidity measurements, or fluorescence metal-sensitive indicators that change color, are well-established, but cannot distinguish between multiple target sequences and may even detect unspecific products [11,12,13].

DNA detection using a lateral flow dipstick involves DNA–DNA hybridization in which free single-strand tag sequences attached to the end of amplicons bind to complementary probes in the dipstick without the need for amplicon denaturation. The reaction can be visualized by the accumulation of colorant [14,15]. Recently, Tian et al. developed a single-stranded tag hybridization chromatographic printed-array strip (STH-PAS) genotyping method [16]. Multiple targets amplified using biotin-labeled primers and unique single-stranded sequence-tagged primers are developed on a printed array strip (C-PAS) using streptavidin-coated blue latex beads [17]. In the multiplex LAMP amplification of *Plasmodium *spp. and *Rickettsia *spp. from human blood, this method has been applied for the rapid visualization of signals [18].

In this study, we developed a LAMP assay combined with DNA chromatography to detect five major carbapenemase-producing genes (*bla*_NDM_, *bla*_OXA-48-like_, *bla*_IMP_, *bla*_KPC_, and *bla*_VIM_) and evaluated the method using clinical strains isolated from patient samples. 

## 2. Materials and Methods

### 2.1. Bacterial Isolates

A total of 73 bacterial collections with 64 *Enterobacterales* and 7 *Pseudomonas* spp., as well as one *Acinetobacter gerneri* isolate and one *Aeromonas taiwanensis* isolate that harbor five carbapenemases (*bla*_NDM_, *bla*_OXA-48-like_, *bla*_IMP_, *bla*_KPC_, and *bla*_VIM_) verified by sequencing were used for this study (Table 1). Among these collections, 8 had two or three carbapenemase genes in each sample. Genomic DNA was extracted from the bacterial collections using a QIAamp^®^ DNA Mini kit (QIAGEN, Hilden, Germany) following the manufacturer’s instructions. 

### 2.2. LAMP Primer Design

The LAMP primer sets for detecting the carbapenemase gene groups *bla*_NDM_, *bla*_OXA-48-like_, *bla*_IMP_, *bla*_KPC_, and *bla*_VIM_ target a common region of each group and were designed using Primer Explorer V5 software (http://primerexplorer.jp/v5_manual/index.html (accessed on 18 April 2019); Eiken Chemical, Tokyo, Japan). The *bla*_NDM_ and *bla*_OXA-48-like_ groups included 19 nucleotide sequences each for *bla*_NDM-1_ to *bla*_NDM-19_ and *bla*_OXA-48_ to *bla*_OXA-567_, respectively. The *bla*_IMP_ group included 65 nucleotide sequences for *bla*_IMP-1_ to *bla*_IMP-67_ and the *bla*_KPC_ group had 29 nucleotide sequences for *bla*_KPC-2_ to *bla*_KPC-32_. The *bla*_VIM_ group had 52 nucleotide sequences for *bla*_VIM-1_ to *bla*_VIM-54_ (Table 2). Primers for conventional LAMP and LAMP DNA chromatography to detect each group are shown in Table 3 and Table 4, respectively.

### 2.3. Conventional LAMP Method

The LAMP reaction was performed using a Loopamp^®^ DNA amplification kit D (Eiken Chemical) following the manufacturer’s instructions. The LAMP reaction was performed in a total volume of 25 μL, comprising 1 μL target DNA template, 1.6 μM FIP and BIP primers, 0.2 μM of each outer primer (F3 and B3), and 0.8 μM of each loop primer (LoopF and LoopB).

The reaction was performed at 65 °C for 40 min using the real-time turbidity-measuring device Loopamp EXIA (Eiken Chemical). At the end of the reaction period, the enzyme mixture was inactivated with a 5-min incubation at 80 °C. Amplicons from the LAMP reaction were detected using turbidity measurements collected every minute of the reaction period using the Loopamp EXIA instrument. 

### 2.4. Development of the Multiplex LAMP DNA Chromatography Detection Method

**Figure 1:**
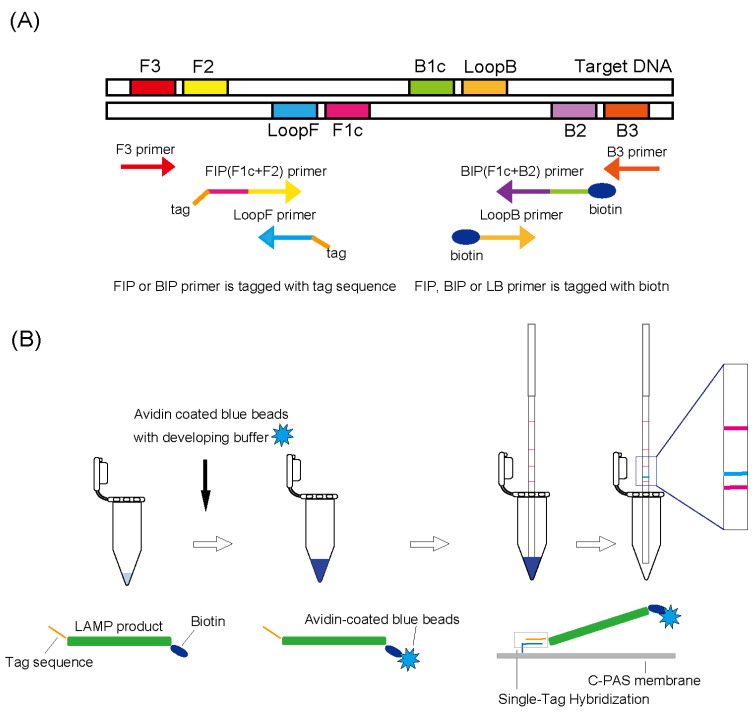
Schematic representation of LAMP DNA chromatography. (**A**) LAMP amplification is performed using six primers (FIP, BIP, F3, B3, LoopF, and LoopB). The FIP and BIP primers are labeled with a tagged sequence or biotin. The LoopB primer is tagged with biotin. (**B**) DNA products are labeled with a tagged sequence and biotin during LAMP amplification. The biotin-labeled LAMP products are labeled upon binding to avidin-coated blue beads. LAMP products labeled with blue beads and a tag sequence at the 5’ end are trapped by oligonucleotides carrying a sequence complementary to the tag sequence and printed on a strip membrane to allow visualization (blue line).

**Figure 2:**
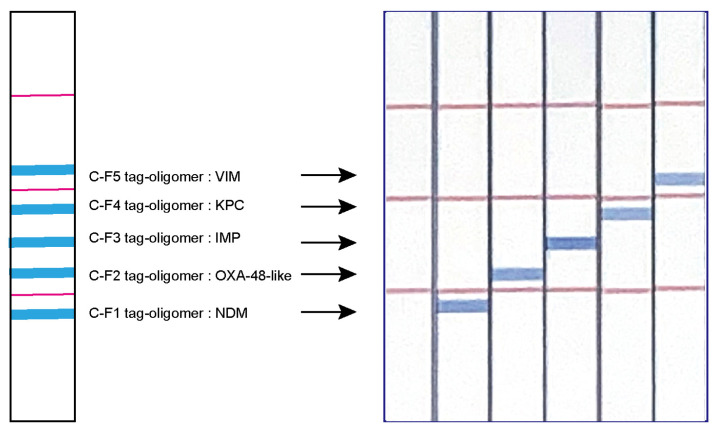
Design of a LAMP DNA chromatography strip to allow simultaneous detection of five different carbapenemase genes. Oligonucleotides having complementary sequences to each of the tag sequences were first printed on a DNA chromatography strip. Tag sequences present in the LAMP products hybridize to complementary sequences carried by oligonucleotides coated onto the DNA chromatography strip. Blue lines indicate presence of the corresponding gene.

We used the STH-PAS system to develop a multiplex detection method to identify five carbapenemase gene groups. Dipstick DNA chromatography strips (C-PAS) and reagents were obtained commercially (TBA, Sendai, Japan). For LAMP DNA chromatography, the 5’ end of FIP or BIP primers were labeled with a tagged sequence, and the 5’ end of FIP, BIP, or LoopB primers were labeled with biotin (Figure 1A). The LAMP amplification was performed in a heat block at 65 °C for 40 min to generate LAMP products having a tagged sequence and biotin. The C-PAS F8 membrane strip (TBA) was inserted into a 21 µL reaction mixture containing 1 µL of LAMP product, 10 µL of developing solution (TBA), 9 µL distilled water, and 1 µL of avidin-coated blue latex beads (TBA). Five complementary oligonucleotide tags (c-tags 1-5) were immobilized on the individual detection line of the C-PAS. LAMP products labeled with latex blue beads were trapped by oligonucleotides having sequences complementary to the tag sequences printed on the strip, and the avidin-coated blue latex beads then bind to biotin to generate a blue line (Figure 1B). All of the samples with carbapenemase genes showed positive blue lines at the position corresponding to each gene group on the C-PAS (Figure 2).

### 2.5. Determination of Detection Limit

The concentration of the extracted genomic DNA was determined three times each using a NanoDrop 2000C (Thermo Fisher Scientific Inc, Waltham, MA, USA), a widely used spectrophotometer, for *E. coli* including *bla*_NDM-1_, *bla*_OXA-48_ or *bla*_KPC-2_, *K. pneumoniae* including *bla*_IMP-1_, and *C. freundii* including *bla*_VIM-2_. The carbapenemase genes of clinical isolates were verified by sequencing and aligned consensus sequences: NDM-1 (accession no. FN396876), OXA-48 (accession no. AY236073), KPC-2 (accession no. AY034847), IMP-1 (accession no. KX452681), VIM-2 (accession no. AF191564) (data not published). A 10-fold dilution series of genomic DNA was used to evaluate the detection limits of the PCR method as well as those for conventional LAMP and LAMP DNA chromatography.

## 3. Results

### 3.1. Detection Limit

We first determined the detection limits for identifying *E. coli* including *bla*_NDM-1_, *bla*_OXA-48_, and *bla*_KPC-2_*, K. pneumoniae* including *bla*_IMP-1_, and *C. freundii* including *bla*_VIM-2_ by the conventional LAMP method, LAMP DNA chromatography, and PCR (Table 5). We then compared the detection limits of conventional LAMP and LAMP DNA chromatography with those of the PCR method that was previously reported for genomic DNA extracted from the five abovementioned bacteria [19,20,21,22]. The LAMP method and LAMP DNA chromatography showed higher detection limits for identifying the five different genes (*bla*_NDM-1_, *bla*_OXA-48_, *bla*_IMP-1_, *bla*_KPC-2_, and *bla*_VIM-2_) than the PCR method. In addition, LAMP DNA chromatography had similar or higher sensitivity than the conventional LAMP method.

### 3.2. Sensitivity and Specificity of the Conventional LAMP Method and LAMP DNA Chromatography to Detect Carbapenemase Genes

The sensitivity and specificity of the conventional LAMP method using a real-time turbidity-measuring device and the LAMP DNA chromatography method were evaluated using 73 bacterial collections containing 78 carbapenemase genes: sixteen *bla*_NDM_, nineteen *bla*_OXA-48-like_, twenty *bla*_IMP_, fourteen *bla*_KPC_, and nine b*la*_VIM_. The results were compared with information for carbapenemase genes of a bacterial collection that was previously characterized by sequencing. Compared to the conventional LAMP method, LAMP DNA chromatography showed similar or higher sensitivity and specificity for identifying five different carbapenemase genes (Table 6).

### 3.3. Multiplex Detection of LAMP Products Using a Single DNA Chromatography Strip

**Figure 3:**
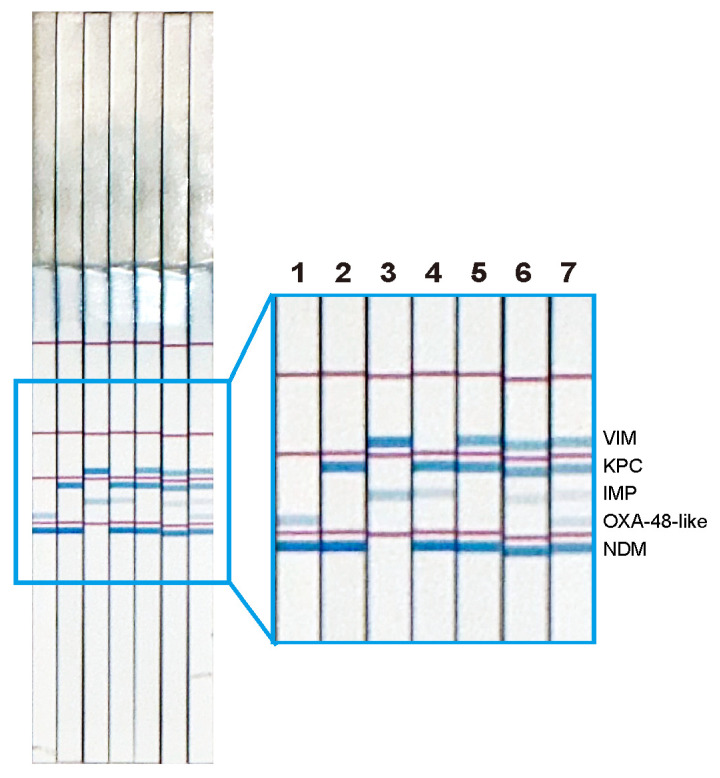
Successful detection of multiple LAMP products using a single DNA chromatography strip. LAMP products were randomly selected and mixed in a test tube, then simultaneously visualized on a single DNA chromatography strip. Lane 1: *bla*_NDM_+ *bla*_OXA-48-like_; Lane 2: *bla*_NDM_ + *bla*_KPC_; Lane 3: *bla*_IMP_ + *bla*_VIM_; Lane 4: *bla*_NDM_ + *bla*_IMP_ + *bla*_KPC_; Lane 5: *bla*_NDM_ + *bla*_KPC_ + *bla*_VIM_; Lane 6: *bla*_NDM_ + *bla*_IMP_ + *bla*_KPC_ + *bla*_VIM_; and Lane 7: *bla*_NDM_ + *bla*_OXA-48-like_ + *bla*_IMP_ + *bla*_KPC_ + *bla*_VIM_.

LAMP reactions for five carbapenemase genes in each test tube were performed in a heat block at 65 °C for 40 min. Then, between two and five randomly selected different LAMP products were mixed in a single test tube for the simultaneous detection of multiple genes. A C-PAS F8 membrane strip was inserted into a 21 µL reaction mix containing 1 µL of the mixed LAMP products, 10 µL developing solution, 9 µL distilled water, and 1 µL avidin-coated latex beads. Two to five different LAMP amplicons were detected simultaneously in a single tube without cross-reactions (Figure 3), indicating that LAMP DNA chromatography can simultaneously detect five different carbapenemase genes on a single DNA chromatography strip.

## 4. Discussion

In this study, we developed a LAMP DNA chromatography method to detect five different carbapenemase gene groups (*bla*_NDM_, *bla*_OXA-48-like_, *bla*_IMP_, *bla*_KPC_, and b*la*_VIM_). This method is rapid and requires no expensive equipment or specialized technical expertise. This newly developed method exhibited a superior detection limit compared to previously reported PCR methods, as well as high sensitivity and specificity for identifying the five carbapenemase genes. This new method can be a valuable CPE diagnostic tool in regions where expensive equipment is not available for diagnosis and infection control. 

A decade ago carbapenemases were not commonly produced by *Enterobacterales*, but the recent rapid emergence and worldwide dissemination of CPE raises serious public health concerns [3]. Evidence suggests that patients who are infected by carbapenem-resistant pathogens have increased morbidity and mortality [6]. CPE can transfer resistance genes to other pathogens in hospital environments to cause large mono- or multiclonal hospital outbreaks. Thus, the early detection of specific carbapenemase-producing genes is critical to reduce the risk of mortality, hospital outbreaks, and associated hospital costs as well as to select optimal treatments. 

Several methods like the modified-Hodge test, ethylenediaminetetraacetic acid inhibition test, MALDI-TOF MS, Carba NP test, and carbapenem inactivation methods have been used to detect carbapenemase activity [23,24,25,26,27]. However, some phenotypic tests require expensive instruments and special culturing conditions that are time-consuming and not available in all countries. Several LAMP-based methods involving real-time turbidimeter measurements have been reported for the detection of *bla*_NDM_, *bla*_OXA-48-like_, *bla*_IMP_, *bla*_KPC_, and b*la*_VIM_ [28,29]. Although real-time turbidimeters offer high sensitivity, these instruments are costly. Furthermore, these techniques have not yet been applied for simultaneous multiple-target detection methods because individual LAMP amplicons derived from each target sequence cannot be distinguished. In the newly developed LAMP DNA chromatography method reported here, each tag sequence is assigned to a LAMP target sequence, which allows the simple identification of LAMP amplicons based on positions of blue bands on the DNA chromatography strip. Multiple amplified targets can be differentiated and visualized without sophisticated equipment, such as a thermocycler. The LAMP DNA chromatography method requires minimum resources—only a heating block is needed for amplification—and thus, this approach is suitable for use in resource-limited countries. Furthermore, LAMP DNA chromatography has a superior detection limit, as well as high sensitivity and specificity for identifying five carbapenemase genes. 

Thus, this method can be made widely available worldwide for point of care testing. However, care must be taken not to open the LAMP test tube after amplification to prevent the spread of LAMP products. The LAMP DNA chromatography method should therefore ideally be performed in separate spaces and rooms [13]. In this respect, we are already developing a novel all-in-one diagnostic kit that combines the LAMP method and DNA chromatography in a completely sealed device.

## 5. Conclusions

In conclusion, we developed a LAMP DNA chromatography method to detect five different carbapenemase gene groups (*bla*_NDM_, *bla*_OXA-48-like_, *bla*_IMP_, *bla*_KPC_, and *bla*_VIM_). This newly developed method can be a valuable rapid diagnostic test for selecting appropriate treatments for patients infected with CPE and for broad-ranging infection control, particularly in low- or middle-income countries.

## Figures and Tables

**Table 1:** Bacterial strains used in this study.

Species (n)	Carbapenemase Genes (n)
Carbapenemase producers	
*Escherichia coli* (34)	*bla*_NDM-1_ (3)*, bla*_NDM-5_ (5), *bla*_NDM-7_ (2),*bla*_OXA-1_ (1), *bla*_OXA-1-like_ (2), *bla*_OXA-48_ (5), *bla*_OXA-181_ (8), *bla*_KPC-2_ (6), *bla*_KPC-19_ (1), *bla*_IMP-1_ (2), *bla*_IMP-6_ (2), *bla*_VIM-2_ (1)
*Klebsiella pneumoniae* (22)	*bla*_NDM-1_ (2), *bla*_OXA-48_ (1), *bla*_OXA-232_ (3), *bla*_OXA-244_ (2), *bla*_KPC-2_ (3), *bla*_KPC-19_ (1), bla_IMP-1_ (6), *bla*_IMP-6_ (5), *bla*_VIM-2_ (1)
*Citrobacter freundii* (5)	*bla*_NDM-7_ (1), *bla*_KPC-2_ (1), *bla*_VIM-2_ (2), *bla*_IMP-1_ (1)
*Enterobacter cloacae* (2)	*bla*_KPC-2_ (1), *bla*_IMP-6_ (1)
*Pseudomonas aeruginosa* (2)	*bla*_IMP-44_ (1), *bla*_VIM-2_ (1)
*Pseudomonas alcaligenes* (1)	*bla*_KPC-2_ (1), *bla*_VIM-2_ (1)
*Pseudomonas mendocina* (2)	*bla*_VIM-2_ (2)
*Pseudomonas putida* (2)	*bla*_NDM-1_ (1), *bla*_VIM-2_ (1), *bla*_IMP-4_ (1)
*Serratia marcescens* (1)	*bla*_OXA-48_ (1)
*Acinetobacter gerneri* (1)	*bla*_NDM-1_ (1), *bla*_IMP-26_ (1)
*Aeromonas taiwanensis* (1)	*bla*_NDM-5_ (1)

**Table 2:** Target carbapenemase genes.

Carbapenemase Gene Group	Genotype	GenBank Accession Numbers
*bla*_NDM_	*bla*_NDM-1_ to *bla*_NDM-19_	FN396876, JF703135, JQ734687, JQ348841, JN104597, JN967644, JX262694, AB744718, KC999080, KF361506, KP265939, AB926431, LC012596, KM210086, KP735848, KP862821, KX812714, KY503030, MF370080.1
*bla*_OXA-48-like_ (*n* = 65)	*bla*_OXA-48_, *bla*_OXA-162_, *bla*_OXA-163_, *bla*_OXA-181_, *bla*_OXA-204_, *bla*_OXA-232_, *bla*_OXA-244_, *bla*_OXA-245_, *bla*_OXA-247_, *bla*_OXA-370_, *bla*_OXA-405_, *bla*_OXA-436_, *bla*_OXA-439_, *bla*_OXA-484_, *bla*_OXA-505_, *bla*_OXA-517_, *bla*_OXA-519_, *bla*_OXA-566_, *bla*_OXA-567_	AY236073, HM015773, HQ700343, JN205800, JQ809466, JX423831, JX438000, JX438001, JX893517, KF900153, KM589641, KT959108, KP727573, KR401105, KU531433, KU878974, KX349732, MF099636, MG062673
*bla*_IMP_ (*n* = 65)	*bla*_IMP-1_ to *bla*_IMP-35_, *bla*_IMP-37_ to *bla*_IMP-56, _ *bla*_IMP-58_ to *bla*_IMP-67_	KX452681, AJ243491, AB010417, AF445082, AF290912, AB188812, AF318077, AF322577, AY033653, AB074433, AB074437, NG_049175, AJ550807, AY553332, AY553333, AJ584652, AJ512502, EF184215, EF118171, AB196988, AB204557, DQ361087, DQ417222, EF192154, EU541448, GU045307, JF894248, JQ407409, HQ438058, DQ522237, KF148593, JQ002629, JN848782, AB700341, JF816544, JX131372, HQ875573, MK507818, AB753457, AB753458, AB753456, AB777500, AB777501, KJ510410, KM087857, KP681694, LC031883, LC055762, HM106457, KU052795, KU299753, KU351745, KU647281, KX196782, LC159227, KX462700, KX753224, KX821663, KX949735, KY315991, LC190726, MF281100
*bla*_KPC_ (*n* = 29)	*bla*_KPC-2_ to *bla*_KPC-19_, *bla*_KPC-2_ to *bla*_KPC-32_	AY034847, AF395881, AY700571, EU400222, EU555534, EU729727, FJ234412, FJ624872, GQ140348, HM066995, HQ641421, HQ342889, JX524191, KC433553, KC465199, KC465200, KP681699, KJ775801, LN609376, KM379100, KR052099, KU216748, KX619622, KX828722, KY282958, KY563764, NG_054685, NG_055494, NG_055495
*bla*_VIM_ (*n* = 52)	*bla*_VIM-1_ to *bla*_VIM-20_, *bla*_VIM-23_ to *bla*_VIM-54_	Y18050, AF191564, AF300454, AY135661, DQ023222, AY16502, AJ536835, AY524987, AY524988, AY524989, AY605049, DQ143913, EF577407, AY635904, EU419745, EU419746, EU118148, AM778091, FJ822963, GQ414736, GQ242167, HM855205, HM750249, FR748153, HQ858608, JF900599, JX311308, JN129451, JN982330, JN676230, JX258134, JX013656, JX982634, JX982635, JX982636, KC469971, KF131539, HG934765, KP771862, KP071470, KP096412, KP681696, KP681695, KP749829, KT954134, KT964061, KU663374, KU663375, KU746270, KX349731, KX788872, KY508061

**Table 3:** Nucleotide sequences of conventional LAMP primers to detect *bla*_NDM_*, bla*_OXA-48-like_*, bla*_IMP_, bla_KPC_, and *bla*_VIM_.

Gene	Primer	Sequence (5′ → 3′)
*bla*_NDM_	F3	TCGATACCGCCTGGACC
	B3	TCGACAACGCATTGGCATA
	FIP	GCGCGACCGGCAGGTTGATGATGACCAGACCGCCCAG
	BIP	CGGTGGTGACTCACGCGCAAGTCGCAATCCCCGCC
*bla*_OXA-48-like_	F3	AATAGCTTGATCGCCCTC
	B3	CCATAATCGAAAGCATGTAGC
	FIP	GATTCCAAGTGGCGATATCGCGGCGTGGTTAAGGATGAAC
	BIP	TAATCACCGCGATGAAATATTCAGTCTTGCTCATACGTGCCTC
	LF	GCGTCTGTCCATCCCACTTA
	LB	GAATTTGCCCGCCAAATTGG
*bla*_IMP_	F3	GCGTTGTTCCTAAACATGG
	B3	TTGTTAATTCAGATGCATACGT
	FIP	TCCACAAACCAAGTGACTAACTTTTTGGTTCTTGTAAATGCTGAGG
	BIP	CGTGGCTATAAAATAAAAGGCAGCAGATCGAGAATTAAGCCACTC
	LF	AGTATCTTTAGCCGTAAATGGAGTG
	LB	CGACAGCACGGGCGGAATA
*bla*_KPC_	F3	ACTCCGCCATCCCAAGC
	B3	TCCGACTGCCCAGTCTG
	FIP	TGCAGAGCCCAGTGTCAGTTTTGATGCGCGCGATACCTC
	BIP	GCAGCGGCAGCAGTTTGTTGCGGATGCGGTGGTTGC
	LF	GTAAGCTTTCCGTCACGGCG
	LB	TTGGCTAAAGGGAAACACGACC
*bla*_VIM_	F3	AATTCCGGTCGGAGAGGT
	B3	AAAGTGCGTGGAGACTGC
	FIP	CATTGGACGGGTAGACCGCGCCAGATTGCCGATGGTGT
	BIP	TGATTGATACAGCGTGGGGTGCACGGGAAGTCCAATTTGCT
	LF	CCATCAAACGACTGCGTTGC

**Table 4:** Nucleotide sequences of LAMP DNA chromatography primers to detect *bla*_NDM_, *bla*_OXA-48-like_, *bla*_IMP_, *bla*_KPC_, and *bla*_VIM_.

Gene	Primer	Sequence (5′→3′)
*bla*_NDM_	F3	TCGATACCGCCTGGACC
	B3	TCGACAACGCATTGGCATA
	FIP-biotin ^a^	GCGCGACCGGCAGGTTGATGATGACCAGACCGCCCAG
	BIP-tag ^b^	CGGTGGTGACTCACGCGCAAGTCGCAATCCCCGCC
*bla*_OXA-48-like_	F3	AATAGCTTGATCGCCCTC
	B3	CCATAATCGAAAGCATGTAGC
	FIP-tag ^b^	GATTCCAAGTGGCGATATCGCGGCGTGGTTAAGGATGAAC
	BIP	TAATCACCGCGATGAAATATTCAGTCTTGCTCATACGTGCCTC
	LF	GCGTCTGTCCATCCCACTTA
	LB-biotin ^a^	GAATTTGCCCGCCAAATTGG
*bla*_IMP_	F3	GCGTTGTTCCTAAACATGG
	B3	TTGTTAATTCAGATGCATACGT
	FIP-tag ^b^	TCCACAAACCAAGTGACTAACTTTTTGGTTCTTGTAAATGCTGAGG
	BIP-biotin ^a^	CGTGGCTATAAAATAAAAGGCAGCAGATCGAGAATTAAGCCACTC
	LF	AGTATCTTTAGCCGTAAATGGAGTG
	LB	CGACAGCACGGGCGGAATA
*bla*_KPC_	F3	ACTCCGCCATCCCAAGC
	B3	TCCGACTGCCCAGTCTG
	FIP-tag ^b^	TGCAGAGCCCAGTGTCAGTTTTGATGCGCGCGATACCTC
	BIP-biotin ^a^	GCAGCGGCAGCAGTTTGTTGCGGATGCGGTGGTTGC
	LF	GTAAGCTTTCCGTCACGGCG
	LB	TTGGCTAAAGGGAAACACGACC
*bla*_VIM_	F3	TGGTCGCATATCGCAACG
	B3	GCCCGAAGGACATCAACG
	FIP-tag ^b^	GCACCCCACGCTGTATCAATCAGGTCTACCCGTCCAATGGT
	BIP-biotin ^a^	AACACAGCGGCACTTCTCGCTGAAAGTGCGTGGAGACTG
	LF	GCAACTCATCACCATCACGGACAA
	LB	GCAAATTGGACTTCCCGTAACG

^a^ Biotin was added to the 5′ end; ^b^ Tag sequence was added to the 5′ end.

**Table 5:** Detection limit of PCR, conventional LAMP method, and LAMP DNA chromatography.

Bacteria Including Carbapenemase Gene	Detection Limit (pg/µL)		
	PCR	Conventional LAMP	LAMP DNA Chromatography
*E. coli* including *bla*_NDM-1_	1 × 10^3^	1 × 10^2^	10
*E. coli* including *bla*_OXA-48_	1 × 10^3^	10	1
*K. pneumoniae* including *bla*_IMP-1_	1 × 10^3^	1 × 10^2^	10
*E. coli* including *bla*_KPC-2_	1 × 10^2^	1 × 10^2^	10
*C. freundii* including *bla*_VIM-2_	1 × 10^3^	10	10

Dilution series from 1 × 10^4^ to 1 × 10^−1^ pg/μL was made using 10-fold serial dilutions of genomic DNA extracted from bacteria.

**Table 6:** Sensitivity and specificity of conventional LAMP and LAMP DNA chromatography.

Genotype	Conventional LAMP		LAMP DNA Chromatography	
	Sensitivity	Specificity	Sensitivity	Specificity
*bla*_NDM_	100%	100%	100%	100%
*bla*_OXA-48-like_	100%	100%	100%	98%
*bla*_IMP_	100%	98%	100%	100%
*bla*_KPC_	93%	98%	100%	97%
*bla*_VIM_	100%	100%	100%	100%

## Data Availability

Not applicable.
